# How to use the WHO criteria to focus a hand hygiene campaign

**DOI:** 10.1186/1753-6561-5-S6-P253

**Published:** 2011-06-29

**Authors:** A Jeurissen, S Monsecour, V Cossey, A Schuermans

**Affiliations:** 1 Hospital hygiene and infection control, Uz Gasthuiberg, Leuven, Belgium

## Introduction / objectives

Proper hand hygiene is critical to prevent healthcare associated infections. Therefore, measurement of hand hygiene compliance is an important tool in infection prevention. After multi model hand hygiene campaign (HHC) compliance is known to remain low.

## Methods

Between October and December 2010, after an intensive HHC of ten years in our 1894-bedded tertiary care centre, hand hygiene opportunities (HHO) as defined according to the 5 categories from the WHO were measured by direct observation. Compliance rates (CR) were calculated as the number of hand hygiene activities (HHA) divided by the HHO.

## Results

A total of 1007 HHO were documented: 134 in the protective isolation ward, 113 in the medical ward, 566 in the intensive care unit (ICU), and 161 in the surgical ward, resulting in CRs of 49% (66/134), 51% (58/113), 28% (159/566), and 51% (82/161), respectively. The CR in the ICU was statistically lower (p < 0.001) compared to the other wards and this was due to the absence of hand hygiene after glove use. In all wards except the protective isolation ward, CRs were statistically lower (p < 0.001) before patient contact (WHO indications 1 and 2) than after patient contact (WHO indications 3, 4 and 5).

## Conclusion

Most increase in compliance can be achieved by focusing our new HHC on performing hand hygiene before aseptic tasks.

## Disclosure of interest

None declared.

